# *In vitro* anticancer screening of 24 locally used Nigerian medicinal plants

**DOI:** 10.1186/1472-6882-13-79

**Published:** 2013-04-08

**Authors:** Saudat Adamson Fadeyi, Olugbeminiyi O Fadeyi, Adedeji A Adejumo, Cosmas Okoro, Elbert Lewis Myles

**Affiliations:** 1 Department of Biological Sciences, Tennessee State University, 3500 John A. Merritt Blvd, Nashville, TN 37217, USA; 2 Department of Chemistry, Tennessee State University, 3500 John A. Merritt Blvd, Nashville, TN 37217, USA; 3 Department of Internal Medicine Meharry Medical College, 1005 Dr. D. B. Todd, Jr. Blvd, Nashville, TN 37208, USA; 4 Department of Chemistry and Chemical Biology, Harvard University, 12 Oxford Street, Cambridge, MA 02138, USA; 5 Department of Wildlife and Eco-tourism Management, Federal College of Wildlife Management, Forestry Research Institute of Nigeria, New Bussa, Niger State, Nigeria

**Keywords:** Nigeria, Anti-cancer, Ethnomedicine, Cytotoxic activity

## Abstract

**Background:**

Plants that are used as traditional medicine represent a relevant pool for selecting plant candidates that may have anticancer properties. In this study, the ethnomedicinal approach was used to select several medicinal plants native to Nigeria, on the basis of their local or traditional uses. The collected plants were then evaluated for cytoxicity.

**Methods:**

The antitumor activity of methanolic extracts obtained from 24 of the selected plants, were evaluated *in vitro* on five human cancer cell lines.

**Results:**

Results obtained from the plants screened indicate that 18 plant extracts of folk medicine exhibited promising cytotoxic activity against human carcinoma cell lines. *Erythrophleum suaveolens* (Guill. & Perr.) Brenan was found to demonstrate potent anti-cancer activity in this study exhibiting IC_50_ = 0.2-1.3 μg/ml.

**Conclusions:**

Based on the significantly potent activity of some plants extracts reported here, further studies aimed at mechanism elucidation and bio-guided isolation of active anticancer compounds is currently underway.

## Background

Currently, one in four deaths in the United States is due to cancer [1]. When ranked within age groups, cancer is one of the five leading causes of death amongst both males and females and the single largest cause of death worldwide [1]. By 2015 cancer morbidity may climb to around nine million world-wide. This growing trend indicates deficiency in the present cancer therapies which include surgical operation, radiotherapy and chemotherapy. Since the average survival rates have remained essentially unchanged despite such aggressive treatments, there is a critical need for anti-cancer agents with higher efficacy, and less side effects that can be acquired at an affordable cost.

We suppose that plants are the best alternative, as they provide an inexhaustible pool of efficacious agents for treating disease. Phytochemicals have always been sought after because of their inherent potential to cure disease, as demonstrated by ancient medicinal practices [2-5]. Furthermore, several plants have been shown to be sources of therapeutically important agents, valuable in the treatment of cancer. For instance, there are very effective cancer chemotherapeutic drugs that have been derived from natural origin [6]. These include plant-derived agents, such as the vinca alkaloids vinblastine and vincristine, isolated from the Madagascar periwinkle, *Catharanthus roseus* (L.) G. Don. [7]; paclitaxel (Taxol), originally isolated from the bark of the Pacific yew tree from the Pacific Northwest, *Taxus bre*v*ifolia* Nutt.*,* and the analogue, docetaxel [8]; etoposide and teniposide, derived semisynthetically from epipodophyllotoxin, an epimer of podophyllotoxin, isolated from roots of *Podophyllum* species [9]; and camptothecin, isolated from the bark of *Camptotheca acuminata* Decne., a precursor to the semisynthethetic drugs topotecan (Hycamptin) and irinotecan (Camptosar) [10].

There are estimated to be between 200,000 and 450,000 species of tropical flowering plants within our biosphere, with the greatest plant diversity being found in the moist tropics [11,12]. The approaches for selecting plants to be tested for new bioactive compounds range from random selection to ethnopharmalogical approaches relying on knowledge gained from traditional medicine usage. Traditional medicine occupies a central role in the developing nations [13].

Although there have been vast discoveries of potent cytotoxic agents attributed to Asian and Ayurvedic Indian traditional medicine, the need for this study is derived from the fact that much of the medicinal plants found in Africa are unexplored. Drug discovery of African plants is of relevant interest because Africa hosts 57,704 species of the world’s flora [14] and although Africans use over 5000 of their plants for medicinal purposes, the study of African medicinal plants has not been accredited or documented as extensively as the Chinese and Indian herbal medicines [13,15]. The potential of Nigerian flora in particular, as a veritable source for pharmaceuticals and other therapeutic materials has been well documented [16]. In the present study, we performed the preliminary screening of 24 methanolic plant extracts, used in Nigerian folk medicine, to identify plants with cytotoxic activity against five human cancer cell lines.

## Methods

### Collection of plant material and preparation of extracts

Plant materials (the list of plants studied is given in Table 1) were obtained by Mr A. A Adejumo at different times of the year. Specimens were collected from the western part of Nigeria (Lagos, Ogun, Oyo and Osun states) from traditional healers and indigenous herbal merchants. Collected specimens were authenticated by comparison with corresponding herbarium specimens. Some samples have been deposited at the Department of Biological Science, Tennessee State University, Nashville, Tennessee, USA.

**Table 1:** List of plants screened in this study and their report local uses

**Species**	**Family**	**Voucher specimen (Part used)**	**Reported local medicinal uses**	**Extract yield (%)**
Acanthus montanus (Nees) T. Anders	Acanthacease	TVN-A08 (l,r,s)	Syphilis, emetic, urethral discharge, purgative [17]	4.05
Allanblackia floribunda Oliv.	Guttiferae	TVN-A33 (l,b,r,f)	Malaria, dysentery [18]	4.63
Amaranthus spinosus L.	Amaranthaceae	TVN-A04 (l,st)	Diarrhea, dysentery, Gonorrhea [18]	6.76
Bidens pilosa L.	Compositae	TVN-A75 (l,b,st)	Antidiabetic, anaesthetic [19]	8.64
Bryophyllum pinnatum Lam.	Crassulaceae	TVN-A64 (l)	Respiratory tract infections, antibacterial [20]	1.54
Byrsocarpus coccineus Schumach	Connaraceae	TVN-A14 (b,l)	Jaundice, pile, gonorrhea, venereal disease, impotence [21]	5.79
Cajanus cajan L.	Leguminosae	TVN-A09 (l)	Smallpox, chicken pox, malaria [18,22]	4.08
Capsicum frutescens L.	Solanaceae	TVN-A03 (f,s)	Malaria, Fever, dysentery [18]	1.94
Chromolaena odorata (L.) R.M. King & H. Rob.	Rosaceae	TVN-A02 (l,st,r)	Malaria, antimicrobial [18,23]	9.19
Crassocephalum crepidioides (Benth.) S. Moore.	Compositae	TVN-A34 (l,r,s,f)	Indigestion, stomach ache, headache [24]	6.38
Daniellia oliveri Hutch & Dalz.	Leguminosae	TVN-A11 (l)	Backache, headache, antibacterial, yellow fever [25]	5.47
Erythrophleum suaveolens (Guill. & Perr.) Brenan	Leguminosae	TVN-A65 (b)	Poison, cardiac problems, venom intoxication, inflammatory diseases [22]	12.47
Hoslundia opposita Vahl.	Labiatae	TVN-A72 (l)	Abdominal pains, epilepsy, neurotic disorders [26]	5.82
Jatropa curcas L.	Euphorbiaceae	TVN-A19 (l)	Ringworm, eczema, ulcer [18]	1.31
Landolphia dulcis Var.	Barteri Apocynaceae	TVN-A07 (b)	Rheumatism, cough, kidney diseases, antibacterial [27]	5.75
Lannea nigritana (Sc. Elliot) Keay.	Anacardaceae	TVN-A61 (l,b,r)	None	5.09
Ocimum basilicum L.	Lamiaceae	TVN-A10 (l)	Gonorrhea, catarrhal conditions, cough, anthelmintics [28]	9.7
Parkia biglobosa (Jacq.) G.	Don. Leguminosae	TVN-A01 (l)	Malaria, fever [18,23]	3.87
Parkia filicoidea Welw.	Mimosaceae	TVN-A35 (l,st)	None	7.02
Pterocarpus santalinoides DC.	Fabaceae	TVN-A06 (l,st)	Insecticidal, larvicidal [17,29,30]	3. 34
Rauvolfia vomitoria Afzel.	Apocynaceae	TVN-A28 (b)	Sedative/mental disorder, antidiabetic, malaria [19,23]	6.61
Sida acuta Burm. F.	Malvaceae	TVN-A77 (l,st)	Malaria, ulcer, fever [18]	2.47
Tetrapleura tetraptera Taub.	Leguminosae	TVN-A73 (l,r,s,f)	Sickle cell [31]	10.52
Vitex doniana Sw.	Verbenaceae	TVN-A16 (b,r)	Gastroenteritis, diarrhea, antimicrobial [32]	26.75

Plant parts are denoted as follows: 1=leaves, b= Bark st= Stem, s= Seeds, r= Roots.

Plant materials were air dried and separate extracts were made from the leaves, seeds, stems and bark portions, respectively. The methanolic extracts were prepared by immersing portions of the whole plant (200 g) in 500–1000 ml of methanol at room temperature (25°C) and stirred for 6 days. The crude extracts were filtered and the filtrate evaporated using a rotary evaporator. The dissolved constituents were further dried under pressurized vacuum conditions. Stock solutions were prepared by dissolving the dried residue in dimethyl sulphoxide (DMSO). Extract solutions were stored at −20°C until use.

### Cell lines

The six selected cancer cell lines used in this research were derived from human breast adenocarcinoma MCF-7 (ATCC No. HTB-22), BT-20 (ATCC No. HTB-19), BT-549 (ATCC No. HTB-122), prostate adenocarcinoma PC-3 (ATCC No. CRL-1435), acute T cell leukemia Jurkat (ATCC No. TIB-152), and colon adenocarcinoma SW-480 (ATCC No. CCL-228) cells were provided by American Type Culture Collection (Rockville, MD). These cells were grown in RPMI-1640, with the exception of MCF-7, which was grown in Dulbecco’s modified eagle medium (DMEM); all supplemented with 10% heat inactivated fetal bovine serum (FBS), 2 mM L-glutamine, and 1% penicillin-streptomycin. DMEM was also supplemented with 0.01 mg/ml insulin and 1mM sodium pyruvate. Cells were incubated in a 5% CO_2_ humidified incubator at 37°C and passaged bi-weekly.

### Trypan blue exclusion viability assay

Anticancer activity was determined using this assay to measure cell viability [28]. MCF-7, BT-20, BT-549, PC-3, JURKAT and SW-480 cell lines were plated at densities of 1 × 10^5^ and 5 × 10^4^ per well in 12-well and 24-well tissue culture plates, respectively. Cells were incubated at 37°C and 5% CO_2_ for 24 h, after which the cells received treatment with fresh medium supplemented with extracts at concentrations ranging between 0.01 μg/ml-200 μg/ml, for a total volume of 1 ml-2 ml per well in 24 and 12-well plate formats, respectively. The negative controls received fresh medium supplemented with the experimental vehicle, DMSO only. Following 72 h of incubation at 37°C, the cells were trypsinized with 0.25% trypsin-EDTA solution. Cells were then resuspended in phosphate buffer saline (PBS) and stained with 0.4% Trypan blue dye solution (v/v in PBS). Live cells are excluded from the stain while dead cells absorb the stain appearing blue in color under a light microscope enabling the enumeration of viable cells. Cell counts were expressed as mean ± standard deviation (SD), representative of three separate experiments.

### AlamarBlue™ Metabolic assay

This assay incorporates a fluorometric/colorimetric growth indicator based on detection of metabolic activity in which living cells yield a very strong fluorescent product [33]. MCF-7, BT-20, BT-549, PC-3, Jurkat, and SW-480 cell lines were plated at 1 × 10^4^ cells per well in a 96-well black plate and stabilized in medium at 37°C and 5% CO_2_ for 24 h. Following the first 24 h, cells received fresh medium supplemented with test extracts at final concentrations ranging between 0.01 μg/ml-200 μg/ml, in a total volume of 200 μl per well. The negative control received the experimental vehicle DMSO at the same end-concentration of 0.1%. Cytotoxicity as indicated by a reduction in cellular metabolic activity was assayed at 72 h, using AlamarBlue™ (Invitrogen); 20 μl of alamar blue dye (end-concentration of 10%) was added to each well and the plates incubated at 37°C overnight. The plates were then analyzed for fluorescence (F) using the SpectraMax Gemini EM microtiter plate reader at dual wavelengths (560 nm λ excitation, 590 λ nm emission). SoftMax Pro 4.7.1 was used to analyze the data. The following formula was used to calculate the inhibition of cell growth: inhibition (%) = (1 – mean F value of treatment group/mean F value of control) × 100.

### Statistical analysis

Quantitative values obtained per treatment were converted to percentage inhibition. Regression analysis was used to compute the inhibition concentration required to produce a 50% reduction in cell viability (IC_50_). Results were expressed as the mean ± SD of values obtained in triplicate from three independent experiments. Statistical differences between correlated samples were evaluated using Student's *t*-test and noted to be significantly different where p < 0.05. Composite treatments were compared using one-way analysis of variances (ANOVA) and considered significantly different where probability values were found to be equal to or less than 0.05.

## Results and discussion

Samples collected in this study were selected to include plants that have suggested bioactivity on the basis of their non-reported traditional usage as medicines. The following selected plants have been reported to be used in traditional treatments for various diseases and ailments ranging from headache, fever, throat and neck ailments, tonsillitis, cough, bronchitis, asthma, tuberculosis, pneumonia, constipation, hernia, dysentery, diarrhea; diseases due to infections from intestinal worm, filarial; venereal diseases such as gonorrhea, syphilis; diseases of the skin like leprosy, ulcers, sores, boils and other bacterial infections; also systemic diseases, malaria, yellow fever, measles, and small pox; as well as epilepsy, cardiovascular disease, diabetes, high blood pressure, inflammatory conditions and other diseases of liver, kidney, muscle and bone. This resulted in a set of 24 crude methanolic extracts from collected plants shown in Table 1.

The major aim of this study was to identify potential anticancer extracts that were effective, not by virtue of high concentration alone, rather by specific activity demonstrated even at low doses. In order to achieve this aim, the maximum test concentration was set at 200 μg/ml, as the criteria for identifying plants with potent activity within range. Using this criterion, plants with less than 50% inhibitory activity within the test range were excluded from further screening. Although such plants may likely demonstrate greater cytotoxicity at higher concentrations, the focus in this study was limited to plant extracts that caused substantial growth inhibition in a given cell line within the test concentration range of < 200 μg/ml. The assumption was that such activity elicited in the plants’ crude state would be indicative of even greater potent effects in the purified state. As a preliminary means of initially identifying extracts with activity, the effects of treatment were evaluated *in vitro*, in a two dose assay testing lower and upper concentrations of 20 and 200 μg/ml against human carcinoma cell lines. All cytotoxic activity was assessed at 72 h following treatment. The Trypan blue exclusion method and the AlamarBlue™ metabolic assay were utilized to quantify cytotoxic or cytostatic effects.

Overall cytoxicity varied between extracts and between cell lines. Table 2 shows the percent inhibition of treated cells relative to the untreated controls. Initially, plants were screened individually against one or more cancer types from the panel of cell lines consisting of BT-549 (breast carcinoma), BT-20 (breast carcinoma) and PC-3 (prostate carcinoma). Then leads for secondary screening were selected on the basis of inhibition ≥ 50% at concentrations below the set upper limit tested. Plants that were considered moderately active showed cytoxicity ≥ 80% inhibition at 200 μg/ml, however some of these plants were weakly cytotoxic at 20 μg/ml. Very active extracts showed 50% or greater inhibition at 20 μg/ml, these plants were selected for further screening at a wider range of concentrations. Extracts exhibiting ≥ 80% inhibition at 20 μg/ml were considered potent and identified as prime targets for further screening.

**Table 2:** Percent inhibiton values of plants crude extracts on three human cancer cell lines at 20 and 200 μg/ml concentrations

**Species**	**T-549**		**BT-20**		**PC-3**	
	**20 μg/ml**	**200 μg/ml**	**20 μg/ml**	**200 μg/ml**	**20 μg/ml**	**200 μg/ml**
Acanthus montanus	7 ± 5.01	10 ± 3.45	Nd	27 ± 8.38*	Nd	<5
Allanblackia floribunda	66 ± 6.51*	96 ± 3.48*	21 ± 4.55	80 ± 5.38*	13 ± 0.58	92 ± 5.29*
Amaranthus spinosus	16 ± 3.86	<5	8 ± 1.72	32 ± 8.14	Nd	Nd
Bidens pilosa	23 ± 9.50	97 ± 1.63*	Nd	93 ± 1.73*	35 ± 1.08	95 ± 1.53
Bryophyllum pinnatum	24 ± 6.08	96 ± 1.68*	Nd	81 ± 6.51*	Nd	95 ± 1.62*
Byrsocarpus coccineus Bark	20 ± 2.51	100	Nd	93 ± 2.66*	Nd	97 ± 6.15*
Leaves	54 ± 1.76	100	Nd	100	Nd	100
Cajanus cajan	9 ± 1.46	99 ± 0.58*	<5	99 ± 0.17*	23 ± 1.53	100
Capsicum frutescens	<5	10 ± 0.96	<5	39 ± 3.96	11 ± 2.40	41 ± 1.08
Chromolaena odorata	13 ± 4.21	8 ± 3.06	6 ± 0.81	39 ± 2.12	Nd	Nd
Crassocephalum crepidioides	14 ± 2.14	51 ± 1.04*	Nd	10 ± 5.21	Nd	9 ± 1.01
Daniellia oliveri	35 ± 1.55	97 ± 0.21*	<5	66 ± 4.16*	22 ± 10.50	47 ± 5.78
Erythrophleum suaveolens	100	100	100	100	100	100
Hoslundia opposita	19 ± 0.55	96 ± 0.42*	Nd	93 ± 1.67*	Nd	51 ± 8.21*
Jatropa curcas	45 ± 4.01	100	29 ± 0.61	87 ± 1.52*	Nd	Nd
Landolphia dulcis	83 ±1.39	100	Nd	11 ± 1.21	Nd	9 ± 3.11
Lannea nigritana	32 ± 0.32	90 ± 0.17*	Nd	Nd	Nd	21 ± 7.71*
Ocimum basilicum	<5	<5	<5	<5	<5	Nd
Parkia biglobosa	<5	75 ± 3.36*	7 ± 5.13	72 ± 0.61	17 ± 7.21	93 ± 6.03*
Parkia filicoidea	<5	67 ± 3.06*	<5 27 ± 3.70	10 ± 0.70	76 ± 1.53*	
Pterocarpus santalinoides	17 ± 2.52	98 ± 0.45*	<5	11 ± 5.03	<5	17 ± 0.40
Rauvolfia vomitoria	<5 37 ± 1.12	19 ± 0.72	33 ± 1.71	<5	8 ± 4.23	
Sida acuta	91 ± 5.86*	95 ± 3.16*	25 ± 5.03	97 ± 0.57*	27 ± 2.20	97 ± 1.80*
Tetrapleura tetraptera	66 ± 1.38*	100 58 ± 9.13*	100	Nd	Nd	
Vitex doniana Bark	<5	89 ± 1.27*	Nd	55 ± 1.33	Nd	<5
Root	21 ± 1.46	56 ± 2.35*	Nd	61 ± 1.06	Nd	57 ± 1.25

The antiproliferative/cytotoxic effect is expressed in terms of the percent inhibition of cells growth relative to the DMSO control after 72 h exposure to the extracts. Results are expressed as mean ± SD of three rreplicate experiments. Nd = Not determined. *There was a significant difference in cell inhibition in extract-treated cultures compared with DMSO-control in all cell lines (P<0.05).

Based on these criteria, 12 of these plants were categorized as moderately active. There were 6 plants that were considered very active to potent at least against one cancer cell line, these included *Byrsocarpus coccineus* with 54% inhibition at 20 μg/ml (against BT-549), *Allanblackia floribunda* and *Tetrapleura tetraptera* which both exhibited the lowest inhibition of 66% at 20 μg/ml in this category (against BT-549). *Landolphia dulcis* and *Sida acuta* showed between 83% - 91% at 20 μg/ml (against BT-549) and the most potent was *Erythrophleum suaveolens*, exihibiting 100% inhibition at 20 μg/ml*.* In terms of potency between cell lines, *Erythrophleum suaveolens* showed the most consistent activity, causing total growth inhibition of all three cell lines, BT-549, BT-20 and PC-3. The latter were analyzed comprehensively in the second phase of screening. Amongst the plants that were moderately active, there is a trend of selectivity towards BT-549. *Allanblackia floribunda* showed significant variation at 20 μg/ml, inhibiting BT-549 by 66%, but only 21% and 13% of BT-20 and PC-3 respectively. Similarly, amongst the very active plants, *Sida acuta* caused 91% inhibition of BT-549 viability at 20 μg/ml, however the same concentration resulted in only 25% and 27% inhibition of BT-20 and PC-3, respectively.

In Table 3, regression analysis was done to compute the inhibition concentration required to produce a 50% reduction in cell viability (IC_50_) of the plant extracts (R^2^ ≥0.9). The concentration that causes 50% inhibition of the cancer cells by the crude extract of the Nigerian plants species investigated are displayed in Table 3. The solvent extracts of 4 plants showed moderate IC_50_ value ranging from 62.5-177.3 μg/ml (Table 3) against different cancer cell lines. Among them is the bark of *Vitex doniana* with IC_50_ value of 62.5, 84, 89.2 and 171.1 μg/ml (against BT-549, JURKAT, SW-480 and BT-20 respectively) and the root of the same plant showed IC_50_ value of 152.3 and 177.3 μg/ml against BT-20 and PC-3. *Hoslundia opposita* also showed significant activity against BT-549, with an IC_50_ value of 76.4 μg/ml and the bark of *Byrsocarpus coccineus* showed similar activity against JURKAT with IC_50_ value of 65.2 μg/ml (Table 3).

**Table 3:** IC50 (μg/mL) values for the in vitro cytotoxic activity of plants crude extracts on five human cancer cell lines

**Species**	**BT-549**	**BT-20**	**PC-3**	**SW-480**	**JURKAT**
Allanblackia floribunda	14.7 ± 0.23	48.3 ± 2.90	29.4 ± 0.69	57.1 ± 1.16	Nd
Bidens pilosa	43.1 ± 6.09	53.7 ± 2.16*	47.7 ± 2.69*	Nd	75.6 ± 1.06*
Bryophyllum pinnatum	48.2 ± 1.56	82.4 ± 0.17*	48.3 ± 1.05*	Nd	Nd
Byrsocarpus coccineus Bark	24.6 ± 0.99	52.9 ± 4.11*	43.7 ± 1.02*	Nd	65.2 ± 0.87*
Leaves	18.6 ± 4.85	31.3 ± 0.53*	29.1 ± 0.64*	Nd	43.4 ± 1.77*
Cajanus cajan	56.1 ± 10.09	56.8 ± 2.60	50.5 ± 0.76	52 ± 0.53	Nd
Daniellia oliveri	28.1 ± 0.56	153.1 ± 1.56	130.0 ± 0.45	147.0 ± 0.47	Nd
Erythrophleum suaveolens	0.55 ± 0.18	0.50 ± 0.03	1.30 ± 0.14	0.80 ± 0.11	0.20 ± 0.05
Hoslundia opposita	76.4 ± 7.89	56.1 ± 1.57	59.7 ± 8.11	Nd	>200
Jatropa curcas	L. 21.3 ± 0.38	33.4 ± 0.70	>200	>200	>200
Landolphia dulcis	16.3 ± 4.31	>200	>200	>200	Nd
Lannea nigritana	48.2 ± 3.52	Nd	>200	Nd	53.5 ± 0.35*
Parkia biglobosa	100.0 ± 0.67	125.0 ± 2.21	56.1 ± 0.45	136.0 ± 0.81	Nd
Parkia filicoidea	149.0 ± 2.65	>200	94.3 ± 0.50	Nd	Nd
Pterocarpus santalinoides	57.9 ± 0.35	>200	>200	>200	10.2 ± 0.25
Sida acuta	10.3 ± 0.21	41.1 ± 1.05	37.1 ± 0.18	Nd	42.3 ± 0.79
Tetrapleura tetraptera	9.1 ± 1.40	23.1 ± 7.05	Nd	Nd	37.5 ± 5.13*
Vitex doniana Bark	62.5 ± 0.23	171.1 ± 1.33	>200	89.2 ± 6.65	84.0 ± 1.13
Root	44.9 ± 0.10	152.3 ± 1.22	177.3 ± 1.01	45.6 ± 1.35	43.4 ± 0.64

Results are expressed as mean ± SD (μg/mL) of three replicate experiments. Nd = Not determined. There was a significant difference in cell inhibition in extract-treated cultures compared with DMSO-control in all cell lines (P<0.05).

Other cytotoxic activity in the IC_50_ value range from 94.3-153.1 μg/ml against different cancer cell lines were also observed, these include *Parkia biglobosa* (IC_50_ value: 125 μg/ml against BT-20 and 136 μg/ml against SW-480), *Daniellia oliveri* (IC_50_ value: 153.1 μg/ml against BT-20, 130 μg/ml against PC-3 and 147 μg/ml against SW-480) and *Parkia filicoidea* (IC_50_ value: 94.3 μg/ml against PC-3 and 149 μg/ml against BT-549).

*Cajanus cajan* exhibited similar cytotoxic activity against all types of cancer cell lines used, showing IC_50_ value between 50.5-56.1 μg/ml, while the extracts of *Allanblackia floribunda* (IC_50_ = 48.3 μg/ml against BT-20 and IC_50_ = 57.1 μg/ml against SW-480), *Parkia biglobosa* (IC_50_ = 56.1 μg/ml against BT-20), *Pterocarpus santalinoides* (IC_50_ = 57.9 μg/ml against BT-549) and the bark of *Byrsocarpus coccineus* (IC_50_ = 43.7 μg/ml against PC-3 and IC_50_ = 52.9 μg/ml against BT-20) all presented moderate cytotoxic activity. *Sida acuta* exhibited moderate cytotoxic activity against BT-20, JURKAT and PC-3 (IC_50_ = 41.1, 42.3 and 37.1 μg/ml respectively), while the extract of the root of *Vitex doniana* exhibited similar cytotoxic activity against BT-20, SW-480 and JURKAT (IC_50_ = 44.9, 45.6 and 43.4 μg/ml respectively). Other plant extracts that showed moderate cytotoxic activity include *Bidens pilosa* (IC_50_ = 43.1 μg/ml against BT-549, IC_50_ = 53.7 μg/ml against BT-20 and IC_50_ = 47.7 μg/ml against PC-3)*, Lannea nigritana* (IC_50_ = 48.2 μg/ml against BT-549 and IC_50_ = 53.5 μg/ml against JURKAT) *Bryophyllum pinnatum* (IC_50_ = 48.2 μg/ml against BT-549 and 48.3 μg/ml against JURKAT) and *Tetrapleura tetraptera* (IC_50_ = 37.5 μg/ml against JURKAT).

The criteria of cytotoxicity activity for the crude extracts, as established by the American National Cancer Institute NCI) is an IC_50_*<* 30 μg/ml in the preliminary assay [34]. Interestingly, about nine extracts showed similar IC_50_ value close to this concentration. The extract of the leaves of *Jatropha curcas* exhibited cytotoxic activity against human breast adenocarcinoma cells (BT-549) with IC_50_ value of 21.3 μg/ml. Similar cytotoxic activity has been previously reported, where the root of *Jatropha curcas* inhibits the proliferation of human colon adenocarcinoma cells (HT-29, IC_50_ = 18.3 μg/ml) and human hepatocytes cells (Chang liver, IC_50_ = 33.3 μg/ml) [35]. The extract of the leaves of *Byrsocarpus coccineus* also exhibited good cytotoxic activity against human breast adenocarcinoma cell lines BT-549, BT-20 and prostate adenocarcinoma cell line PC-3 (IC_50_ = 18.6, 31.3 and 29.1 μg/ml respectively), while the bark of the same plant showed IC_50_ value of 24.6 μg/ml against BT-549 (Table 3). *Daniellia oliveri, Allanblackia floribunda, Sida acuta* and *Tetrapleura tetraptera* also exhibited promising *in vitro* cytotoxic activity against BT-549 (IC_50_ = 28.1, 14.7, 10.3 and 9.1 μg/ml respectively). It is noteworthy to mention that a weak antitumor activity of *Allanblackia floribunda* has been reported using a potato disc tumor induction assay (13.9% inhibition at 100 μg/disc) [36], while Pieme and coworkers also reported that *Sida acuta* inhibits the proliferation of human hepatoma cells (HepG-2) by 51.62% at 250 μg/ml [37].

Among plants extracts screened on multiple cell lines, four species showed a degree of selectivity. *Jatropha curcas* showed selective activity on breast cancer cell line (IC_50_ = 21.3 μg/ml against BT-549 and IC_50_ = 33.4 μg/ml against BT-20), but no activity was noticed against other types of cancer cell line (PC-3, SW-480, JURKAT). Similar selectivity for BT-549 and T-cell leukemia cell line (JURKAT) was also noticed for *Daniellia oliveri* (IC_50_= 28.1 and 15 μg/ml). *Sida acuta* showed somewhat selectivity against BT-549, with IC_50_ value of 10.3 μg/ml, while a pronounced selective activity was noticed for *Pterocarpus santalinoides* against JURKAT (IC_50_ = 10.2 μg/ml).

The extract from the bark of *Erythrophleum suaveolens* exhibited the most potent activity against all types of cancer cell line used (IC_50_ = 0.2-1.3 μg/ml, Table 3) including breast cancer cells MCF-7 (IC_50_ = 0.63 μg/ml). Earlier studies by Sowemimo and co-workers revealed that the ethanolic extract of *Erythrophleum suaveolens* leaves showed toxicity and mutagenic activity using brine shrimp lethality test [38].

**Figure 1:**
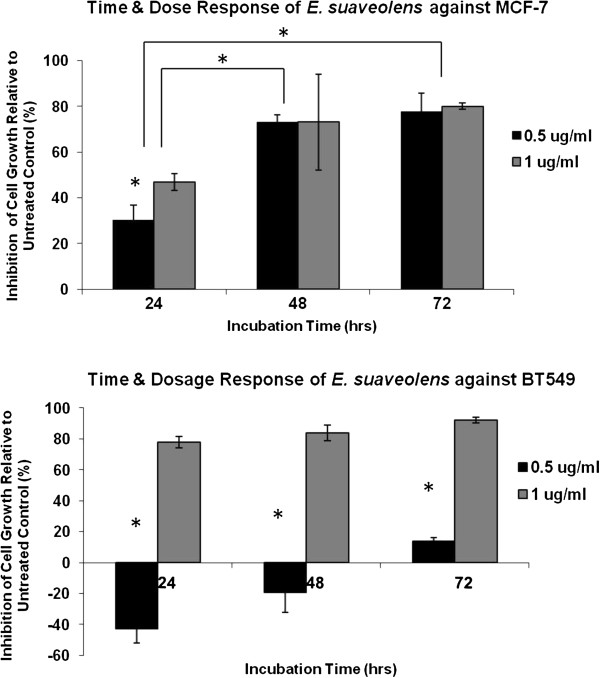
**Time and dose response of *Erythrophleum suaveolens *at 0.5 μg/ml and 1 μg/ml on cell proliferation of MCF-7 and BT-549 breast cancer cell lines.** Cells were plated at 10^4 cells per well in a 96-well plates and treated for 24, 48 and 72 hr. Values are presented as means (n = 3) ± S.D. *Statistical difference (p < 0.05).

In order to gain more insight on the mechanism of *Erythrophleum suaveolens* cytoxicity, it was necessary to evaluate whether the induced anticancer activity was a factor of dosage alone or dosage in correlation with time of exposure (Figure 1).

At low concentrations of *Erythrophleum suaveolens* a decrease of MCF-7 viability to 30% was detected after 48 h of exposure. As shown in Figure 1, with treatment of MCF-7, cytotoxic effects are contingent upon exposure time, which is seen by the drastic increase from 30% inhibition at 24 h to 80% inhibition after 72 h. However, in BT-549 toxicity is dose dependent. No antiproliferation effect of *Erythrophleum suaveolens* is detected within 48 h of exposure to a concentration of 0.5 μg/ml, where in fact greater cell number is observed in treated cells. With increase in dose, at 1 μg/ml, exposure to *Erythrophleum suaveolens* for 24 h is associated with a dramatic fall in BT-549 viability of 80%.

This loss of viability increases only about 10% by 72 h indicating that the effects of *Erythrophleum suaveolens* on BT-549 are fairly rapid within a 24 h period. The data obtained in these preliminary studies provide enough evidence to suggest that *Erythrophleum suaveolens* does in fact contain potent cytotoxic compounds that inhibit tumor cells *in vitro*. In the crude form these active compound(s) may elicit synergistic effects or may even be subdued by the presence of other inactive components.

**Figure 2:**
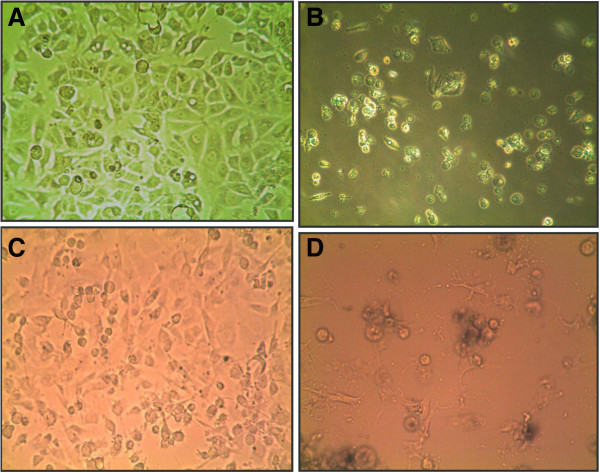
**Microscopic images of *Erythrophleum suaveolens*-treated and untreated cells.** (**A**) DMSO treated BT549 cells after 24 h (top left panel) (**B**) Detachment from culture plate (BT549) after 24 h exposure to *Erythrophleum suaveolens* at its IC_50_ value (0.55 μg/ml), (top right panel) (**C**) DMSO treated BT549 cells after 48 h (bottom left panel) (**D**) Cytostatic effects after 48 h exposure to *Erythrophleum suaveolens* at its IC50 value (0.55 μg/ml), (right panel). All images are magnified at 40×. Images shown are representative of at least five such fields of view per sample and three replicates.

The time response curves reveal a peak in inhibitory activity after 18 h of exposure indicating that within that time frame, enough cellular damage has been inflicted to inhibit approximately 60-80% of cell viability. Physiologically, cells become detached from the base of the culture plate suggesting an interruption of the extracellular matrix and inhibition of cell to cell contact (Figure 2). Analysis with AlamarBlue indicates a complete shutdown of metabolic activity. Furthermore, microscopic comparisons between cells treated with *Erythrophleum suaveolens* and non-treated controls suggest cytostatic effects due the presence of active cellular expansion in controls, which is inhibited in the treated.

## Conclusions

In this study 24 indigenous plants from Southwestern Nigeria were screened for their ability to induce cytotoxicity human cancer cell lines, the results of the study have therefore demonstrated that reliance on ethnomedicinal information as a strategic approach in the selection of native plants is an effective method that yields positive selection of taxonomically diverse leads with very few unfavorable candidates.

In conclusion, this study has demonstrated the successful streamlining of the screening process of bioactive plants with anticancer activity, by eliminating poor candidates on the basis of cytotoxic criterion that takes into consideration effective dosage. Results obtained from folk medicinal plants screened indicate that 18 plant extracts exhibited promising cytotoxic activity against human carcinoma cell lines. *Erythrophleum suaveolens* was found to demonstrate potent anti-cancer activity in this study exhibiting IC_50_ = 0.2-1.3 μg/ml. Among the active extracts, the species with the highest hit rate of demonstrated anticancer activity in this study were from the phylum Leguminosae, which is a large and economically important family of flowering plants which is commonly known as the legume family, pea family, bean family or pulse family. Extensive further analysis on the anticancer properties of *Erythrophleum suaveolens* compared with those of an anticancer drug compound as the positive control is currently underway. Efficacy and mechanisms of action in various normal and cancer cell models *in vitro*, coupled with bio-assay guided purification in order to elucidate active anticancer compound(s) from the crude extract will be reported in due course.

## Competing interests

The authors declare that they have no competing interests.

## Authors’ contributions

SAF and ELM conceived and designed the experiments. SAF performed the cell assay experiments and analyzed the data. OO prepared the crude methanolic extracts. AA obtained and prepared the plants. SAF and OO wrote the paper. CO and ELM supervised the study and revised the manuscript. All authors read and approved the final version of the manuscript to be published.

## Pre-publication history

The pre-publication history for this paper can be accessed here:

http://www.biomedcentral.com/1472-6882/13/79/prepub
